# Engineered nanoparticles for the treatment of Alzheimer’s disease

**DOI:** 10.3389/fphar.2025.1510798

**Published:** 2025-04-03

**Authors:** Jia Jia, Shuang Zhao, Jinghan Zhao, Yun Gao

**Affiliations:** ^1^ Department of Neurological Function Examination, The Fourth Affiliated Hospital of China Medical University, Shenyang, China; ^2^ Endoscopy Center, The Fourth Affiliated Hospital of China Medical University, Shenyang, China; ^3^ Fifth Department of General Surgery, The Fourth Affiliated Hospital of China Medical University, Shenyang, China; ^4^ Second Department of General Surgery, The Fourth Affiliated Hospital of China Medical University, Shenyang, China

**Keywords:** nanoparticles, Alzheimer’s disease, blood-brain barrier, nerve regeneration, β amyloid

## Abstract

Alzheimer’s disease (AD) is one of the most common diseases characterized by neurodegeneration and is becoming a major public health problem worldwide. AD is manifested mainly by progressive impairments in cognition, emotion, language and memory in the elderly population. Many treatment strategies have been explored for decades; however, there is still no effective way to address the root cause of AD pathogenesis, only to target symptoms to improve patient cognitive outcomes. Intracerebral administration is difficult because of the challenges posed by the blood‒brain barrier (BBB). NPs are materials with sizes between 1 and 100 nm that can improve biocompatibility, extend the half-life, transport macromolecules, be delivered across the BBB to the central nervous system, and exhibit good targeting capabilities. NPs can provide new ideas for the treatment of AD in terms of their antiaging, antineuroinflammatory, antioxidative, and nerve repair-promoting effects. In this manuscript, we first describe the relationship between AD and the BBB. Second, we introduce the application of nanoparticles for AD treatment. Finally, we summarize the challenges faced by nanoparticles in the treatment of AD.

## 1 Introduction

Alzheimer’s disease (AD) is a neurodegenerative disease characterized by progressive and irreversible degeneration of the brain, resulting in cognitive impairment and memory decline as the main clinical symptoms (Graff-Radford et al., 2021). The onset of AD usually involves excessive accumulation of β-amyloid (Aβ) and hyperphosphorylated tau protein in the brain, which ultimately leads to neuronal death and cognitive decline (Perneczky et al., 2024). AD is considered to be the most common and challenging mental disorder in society as a whole (Huang et al., 2024). As of 2020, approximately 40 million people worldwide are affected by AD, and this number is increasing annually (Zhang et al., 2023). AD is the leading cause of dementia in older adults, accounting for approximately two-thirds of cases in women compared with men (Zhang, 2023; Lopez-Lee et al., 2024). The onset of AD is thought to be age-related, especially after age 65, with studies showing that more than one in 10 people over the age of 65 have AD (Liu Y. et al., 2024). AD causes severe progressive cognitive impairment, neurobehavioral symptoms that are severe enough to have a significant functional impact on daily life, and patients with severe disease often lose independence (Scheltens et al., 2021). AD not only has a considerable social impact but also increases the economic burden on the global healthcare system to be of paramount importance to healthcare systems (Liu N. et al., 2024).

The treatment of AD is a long-term process, and the current medical community mainly uses drugs and psychotherapy to control the progression of the disease and delay the worsening of symptoms (Liu N. et al., 2024). Cholinesterase inhibitors (donepezil, rivastigmine) and glutamate receptor antagonists (memantine) are commonly used in clinical practice to treat cognitive symptoms (Buccellato et al., 2023). In addition, antipsychotics can also reduce symptoms in the treatment of AD. However, medical therapy can only temporarily relieve symptoms and cannot alter the natural history of AD patients (Aili et al., 2023). In recent years, novel antibody therapies targeting Aβ have been able to significantly reduce the production of Aβ and achieve tangible clinical benefits (Cummings et al., 2023). Some small molecule inhibitors, immunotherapies, and Tau-directed therapies also have potential trends in AD treatment (Liu N. et al., 2024). However, the blood‒brain barrier (BBB), which is composed of a network of capillaries that isolate the brain from blood circulation, prevents many drugs from crossing the BBB (Tosi et al., 2016). Drug treatment for AD is still challenging because of the restrictive nature of the blood‒brain barrier (BBB), which hinders the entry of drugs, thus affecting their efficacy and bioavailability (Elmahboub and Elkordy, 2024; Zhong et al., 2022; Rajendran and Krishnan, 2024). Currently, there is no better way to prevent cognitive decline, and treatment for AD is limited.

**SCHEME 1:**
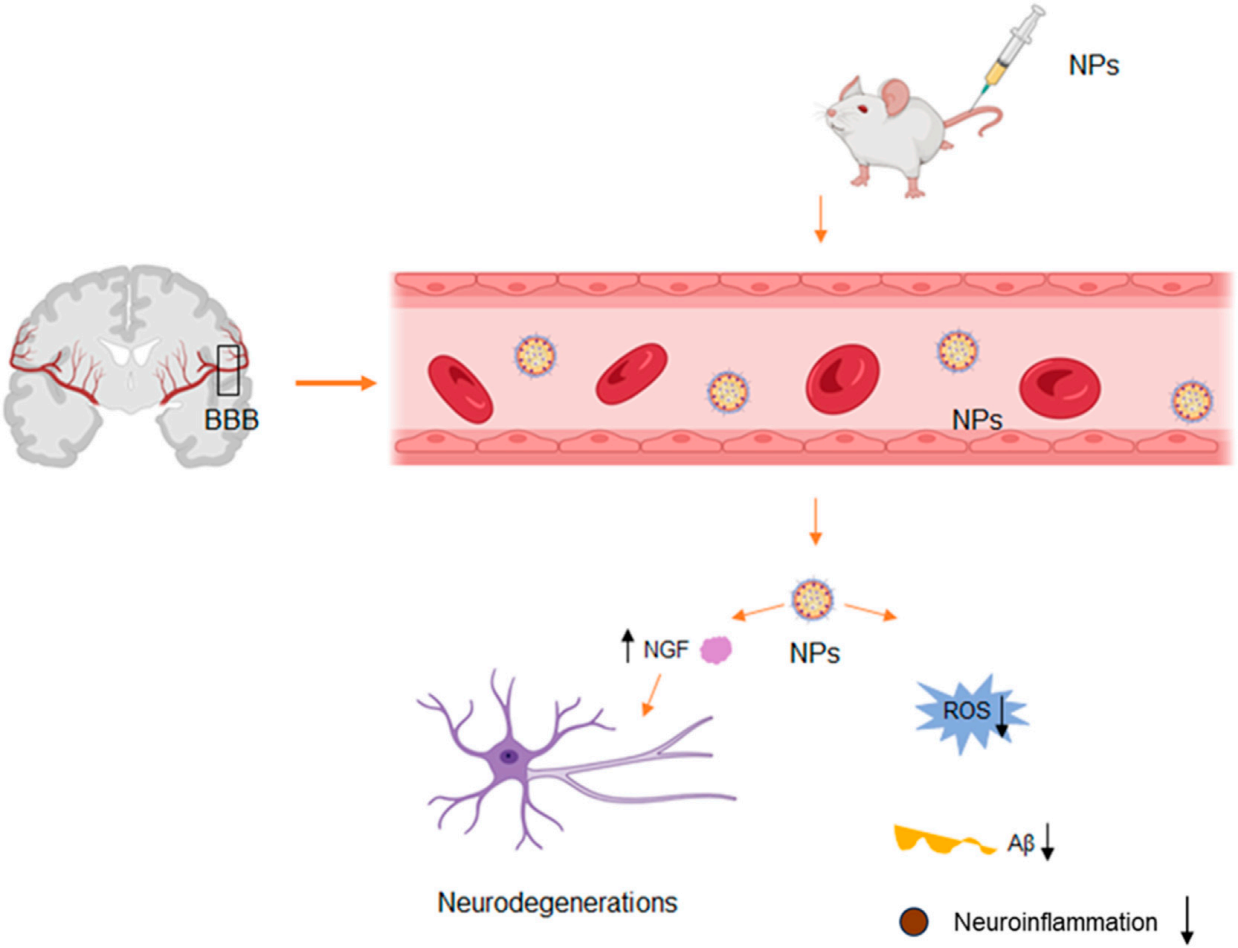
Nanoparticles penetrate the blood-brain barrier to treat Alzheimer’s disease. Nanoparticles are injected into mice through the tail vein and can penetrate the BBB to the lesion site. Subsequently, it causes a decrease in local ROS, reduces nerve inflammation, secretes nerve regeneration factors to promote nerve regeneration, and decreases Aβ.

**SCHEME 2:**
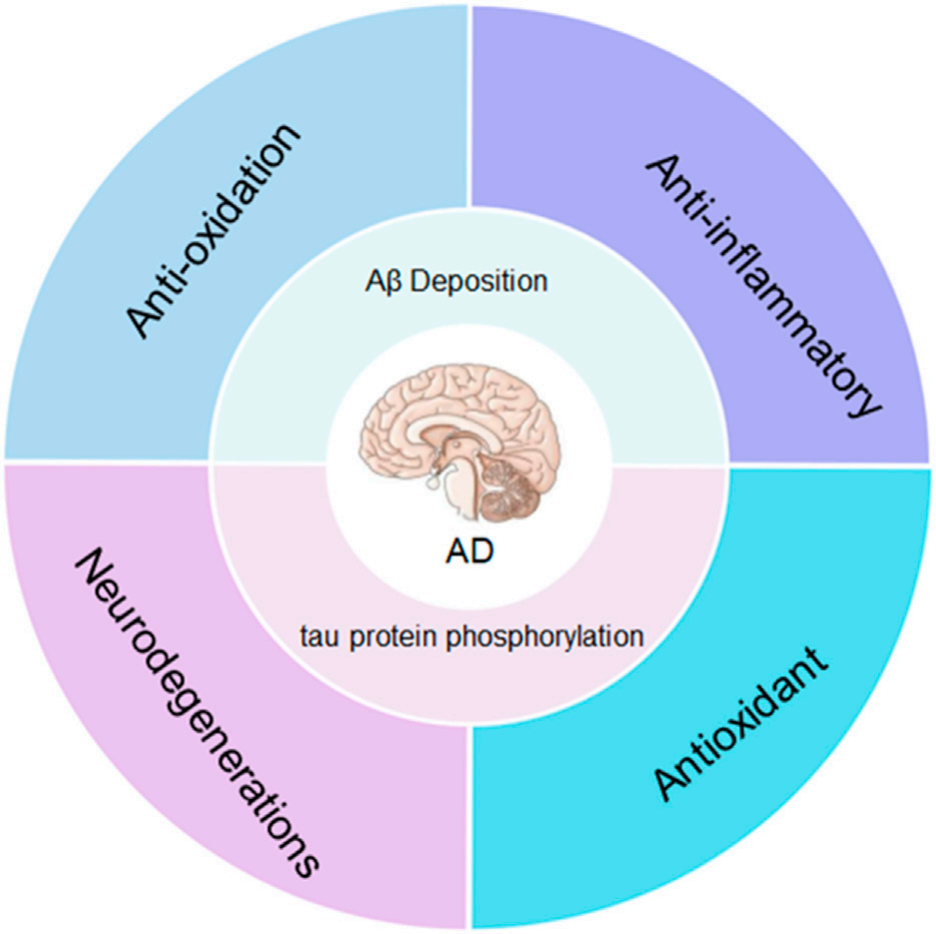
The main pathogenesis of Alzheimer’s disease is the excessive accumulation of amyloid (Aβ) and hyperphosphorylated tau in the brain. Nanoparticles can reduce Aβ and tau protein phosphorylation through anti-aging, anti-inflammatory, antioxidant and promote nerve regeneration.

In recent years, nanomedicine has played an important role in the synthesis and delivery of novel drugs and has been widely used in the biomedical field (Xia et al., 2023a; Xia Y. et al., 2022; Liu et al., 2023). Nanoparticle-loaded drugs can be transported across the BBB to the affected site, increasing the concentration of the drug at the lesion site and thereby improving drug utilization and treatment efficiency (Liu et al., 2023). In this work, we introduce the pathogenesis of AD and introduce the different mechanisms of action of nanoparticles in the treatment of AD (Schemes 1, 2; Table 1), which may provide more opportunities for the therapeutic development of AD in the future.

**TABLE 1:** Engineered nanoparticles for the treatment of Alzheimer’s disease.

Nanopartical	Synthetic materials	Animal models	Nano-properties	Result	References
PLGA nanoparticles	siRNA, PLGA	Mouse	240.4 ± 58.49 nm	Nanoparticle-based delivery of p16ink4a siRNA reduced amyloid plaque formation and the number of aged microglia surrounding the plaque and reversed learning deterioration and spatial memory deficits	Shin et al. (2024)
SSK1-NPs	senescence-specific killer compound 1 (SSK1), β-galactosidase	Mouse	166 ± 5 nm	SSK1-NPs effectively reduce senescent cells in aged mice and are not toxic to normal cells	Cai et al. (2020)
BAP@siRNAs	BA-PDMAEMA, siRNA	Mouse	167.6 nm	The core of NPs goes directly into the cytoplasm, enabling controlled release of siRNAs in a high-ROS environment, downregulating levels of BACE1 and caspase-3, and improving nerve damage	Li et al. (2023)
TR@CPL	Rapamycin and 1‐trifluoromethoxyphenyl‐3‐(1‐propionylpiperidin‐4‐yl) urea (TPPU)	Mouse	—	Administration of nanoliposomes resulted in a significant reduction in amyloid plaque deposition, neuroinflammation, and cognitive impairment	Lin et al. (2024)
PU.1 siRNAs lipid nanoparticles	PU.1 siRNAs	Mouse	—	PU.1 siRNAs lipid nanoparticles achieving up to 92% ± 2% PU.1 transcriptional silencing	Ralvenius et al. (2024)
LNP-R/Aβ	Rapamycin, β-amyloid	Mouse	232.7 ± 84.0	The nanovaccine efficiently delivers rapamycin and Aβ peptides to dendritic cells, alleviates neuroinflammation, and inhibits cognitive impairment in mice	Jung et al. (2023)
LMC-RES	Resveratrol, manganese-doped CeO_2_ nanoparticles	Mouse	The drug load showed 39.8% ± 3.65 for RES	LMC-RES was able to reduce ROS levels, inhibit Aβ-induced neurotoxicity, and protect neurons and significantly improve cognitive deficits of AD mice after drug administration	Hu et al. (2023)
PPA NPs	Prussian blue/polyamidoamine, Angiopep-2	Mouse	Zeta potential of +19 mV	PPA nanoparticles effectively reduce neurotoxic Aβ aggregate and rescue the cognitive functions in APP/PS1 model mice	Zhong et al. (2022)
Zinc oxide nanoparticle	Zinc oxide	Mouse	6.50 ± 0.08 nm	Zinc oxide nanoparticles ameliorate the neurotoxic effects of AD by decreasing IL-1β, TNF-αincreasing glutathione production	Abd Elmonem et al. (2023)
PLGA-curcumin nanoparticles	PLGA, curcumin	Mouse	150 and 200 nm	PLGA nanoparticles are able to destroy amyloid aggregates, exhibit anti-oxidative property and are non-cytotoxic	Mathew et al. (2012)
PLGA@QT NPs	PLGA,quercetin	Mouse	100–150 nm	That injection of PLGA@QT NPs into AD mice ameliorated cognition and memory impairments	Sun et al. (2016)
R@NGF-Se-Se-Ru	Nerve growth factor	Mouse	80 nm	R@NGF-Se-Se-Ru effectively crosses the blood-brain barrier (BBB), reduces Aβ deposition, inhibits Aβ-induced cytotoxicity, and promotes neurite outgrowth	Yuan et al. (2021)
MOF-encapsulated nanozyme	siSOX9, retinoic acid, metal-organic frameworks	Mouse	100–200 nm	MOFs are used to co-deliver siSOX9 and RA to promote neurogenesis	Yu et al. (2020)
PPAR-siSOX9 nanoformulation	PBAE-PLGA-Ag2 S-RA-siSOX9, neprilysin	Mouse	150 nm	Nanopreparation significantly improved cognitive function to a greater degree	Huang et al. (2021)
Naringenin nanoemulsion	Naringenin	Mouse	113.83 ± 3.35 nm	Naringenin-loaded naringenin nanoemulsion significantly attenuated Aβ-induced neurotoxicity and effectively induced nerve repair	Md et al. (2018)
PB NPs/MM	Prussian blue, macrophage membranes	Mouse	—	The enrichment of PB NPs/MM at the lesion site cleared the surrounding inflammation and promoted the nerve repair function in the mouse model of advanced AD	Li et al. (2025)

## 2 Alzheimer’s disease and the blood‒brain barrier

AD is a common chronic neurodegenerative disease characterized by widespread neuronal tangles and amyloid plaques in the brain (De Strooper and Karran, 2016). Aβ and hyperphosphorylated tau protein and neurofilament light chain (NfL) are promising biomarkers for the pathogenesis of AD (Cai et al., 2023). These dense and water-insoluble deposits are deposited in the brain, causing physical damage to surrounding cells (McManus and Latz, 2024). These plasma biomarkers can precisely distinguish AD patients from those who are normal or have other neurodegenerative diseases (Palmqvist et al., 2019). Aβ is formed by sequential cleavage of amyloid precursor proteins by β and γ secretases and is the first protein deposited in the brain (Rajmohan and Reddy, 2017). Aβ deposition triggers a cascade of reactions and progressively leads to Tau pathology, synaptic dysfunction, inflammation, neuronal loss, and eventually dementia (Benilova et al., 2012). With the deposition of large amounts of Aβ, Tau hyperphosphorylation occurs, and these isomers multiply widely to form amyloid plaques and produce neuronal tangles (Cao et al., 2024). These abnormal proteins block neurotransmitter signaling and gradually lead to neurodegeneration (Prajapati et al., 2024). AD is a slowly progressing neurological disease, and autophagy, inflammation, and neurovascular unit dysfunction have been reported to be closely related to the pathogenesis of AD (Prajapati et al., 2024).

The blood‒brain barrier (BBB) is the barrier between the walls of the cerebral capillaries and the plasma formed by glial cells and between brain cells and the plasma and cerebrospinal fluid formed by the choroid plexus; this barrier prevents certain substances (mostly harmful) from entering the brain tissue from the bloodstream (Zapata-Acevedo et al., 2024). The BBB blocks the entry of substances through four main mechanisms: (1) endothelial cells, which are the main functional components of the BBB; (2) enzyme barriers of metabolites; (3) efflux transporter barriers; and (4) immune cells, which form an immune barrier (Gao et al., 2023). More than 98% of small-molecule drugs, including recombinant proteins, peptides, nucleic acids, and monoclonal antibodies, cannot cross the BBB (Chen Y. X. et al., 2020). Therefore, crossing the BBB with drugs is a major challenge in the treatment of AD. There is a strong correlation between microvascular dysfunction in the brain and the pathogenesis of AD (Govindpani et al., 2019). Vascular abnormalities in the AD brain manifest themselves in the early stages of pathology and are characterized by altered vascular morphology, reduced cerebral blood flow (CBF), damage to neurovascular units (NVUs), inflammation of the vasculature, and cerebral amyloid angiopathy, which promote the infiltration of harmful molecules into the brain parenchyma, leading to neuroinflammation and neuronal dysfunction in AD (Lei et al., 2024). The BBB breakdown may precede cognitive decline and neurodegeneration, highlighting the critical need for further exploration of the BBB’s role in AD and its potential as a therapeutic target (Zlokovic, 2008). Because BBB destruction is associated with aging, it does not cause cognitive impairment in patients unless accompanied by inflammation (Alkhalifa et al., 2023). Therefore, the restoration of BBB function is essential for both improving AD pathology and restoring brain function.

## 3 Application of nanoparticles in AD

Nanomaterial delivery strategies have made significant progress in tissue engineering and regenerative medicine (Xia et al., 2023a; Xia Y. et al., 2022). Compared with other drug carriers, nanoparticles exhibit excellent biocompatibility, drug loading capacity, drug delivery efficiency via the BBB, and the ability to target brain damage (Passeri et al., 2022). In addition, nanoparticles have lower immunogenicity and better stability. Nanoparticles have been reported in the treatment of AD.

### 3.1 Anti-aging

Microglia are responsible for phagocytosis of the central nervous system and play important roles in maintaining homeostasis and protecting the brain from infection and damage (Fu et al., 2014). In the AD microenvironment, microglia not only recognize the Aβ peptide and initiate an immune response but also migrate to areas of amyloid deposition (Bolmont et al., 2008). The environment of AD can accelerate the senescence of microglia, which are dysplastic with abnormal loss, enhancement, and distortion of the cytoplasm, leading to a decrease in their phagocytic capacity (Grubman et al., 2021). Xiao et al. used polyD,L-lactic acid-glycolic acid copolymer (PLGA) nanoparticles targeting microglia to deliver p16ink4a siRNA to delay microglial senescence (Shin et al., 2024). PLGA nanoparticles have been reported to target microglia (Shin et al., 2018). Owing to their ability to phagocytose microglia, PLGA nanoparticles release siRNA after phagocytosis into cells, rejuvenating senescent microglia. The particle size of the PLGA nanoparticles was 240.4 ± 58.49 nm. The embedding efficiency was 31.8% ± 0.1%. *In vivo* experiments in rats revealed that the highest release rate was maintained from day 1 to day 5 after intravenous infusion of PLGA nanoparticles. Compared with those in the control group, the number of microglia in the PLGA nanoparticle group was 3.4-fold greater, and the phagocytic capacity of Aβ was increased.

Ji et al. synthesized senescence-specific killer compound 1 (SSK1), which can be hydrolyzed by β-galactosidase to induce apoptosis in senescent cells (Cai et al., 2020). Ji et al. used lipid nanoparticles to load SSK1 to form SSK1-NPs, which significantly reduced the expression of senescence-related genes, induced senescent cell elimination, and reduced lake Aβ accumulation (Ji et al., 2024). SSK1-NPs have a minimum diameter of 166 ± 5 nm and are able to cross the BBB and be deposited in the brain 1 h after intravenous administration. SSK1-NPs are able to release SSK1 when they enter the brain. Studies have shown that SSK1-NPs release 50% of SSK1 in the first 2 h and that 88% of SSK1 is gradually released after 24 h. After 8 weeks of intravenous infusion of SSK1-NPs into rats, SSK1 was induced to eliminate senescent cells in the brains of AD mice via the p38‒p21 axis (Gasek et al., 2021).

Aβ is produced by the amyloid precursor protein (APP), which is proteolytically cleaved by the β-site APP lyase 1 (BACE1) (El Andaloussi et al., 2013). SiRNAs targeting BACE1 can improve Aβ plaques and delay nerve damage in AD (Lane et al., 2018). However, the disadvantage of SiRNAs being unstable and very easy to lyse within cells limits their application (Aly and Waszczak, 2015). Li et al. developed mesenchymal stem cell (MSC)-derived exosomes for SiRNA delivery, while intranasal administration bypassed the BBB to achieve accumulation in the brain (Li et al., 2023). To increase the targeting of exosomes, Li et al. modified exosomes with poly-[(2-methacryloyl)ethyl (benzyl p-borate)dimethylammonium bromide] (BA-PDMAEMA (BAP)) to target ROS (BAP@siRNAs). BAP@siRNAs downregulated the levels of BACE1 and caspase-3 in AD-diseased cells, thereby reducing Aβ plaques and inhibiting neuronal apoptosis. The hydrodynamic diameter of the BAP@siRNAs was approximately 167.6 nm, and the potential was −6.85 mV. BAP@siRNAs are able to release siRNAs in a high-ROS environment. *In vivo* experiments in mice confirmed that BAP@siRNAs could significantly improve the cognitive ability of AD model mice 1 month after intranasal administration.

### 3.2 Reducing neuroinflammation

Although neuroinflammation is often not associated with the occurrence of AD, it can significantly worsen the disease by aggravating Aβ and tau pathologies (Chitnis and Weiner, 2017). Neuroinflammation has increasingly been recognized as a key factor in the pathogenesis of AD (Chen H. Y. et al., 2020). Neuroinflammation severely leads to BBB destruction during AD, increased ROS production, endothelial cell dysfunction, and stromal metalloproteinase (MMP) activation (Alkhalifa et al., 2023). Pro-inflammatory cytokine signaling plays a variety of roles in neurodegeneration and neuroprotection. The induction of pro-inflammatory signaling leads to the release of immune mediators, which affect the function of neurons and lead to cell death (Thakur et al., 2023). Glial cell activation is a central feature of CNS neuroinflammation. These glial cells can produce and release a variety of proinflammatory cytokines, chemokines, and reactive oxygen species (Singh, 2022). Chemokine (C-C motif) ligand 2 (CCL2) is a chemokine produced by astrocytes that can recruit cells expressing CCL2 receptor 2 (CCR2) around AD to the site of injury (Bose and Cho, 2013). 1-Trifluoromethoxyphenyl-3-(1-propionylpiperidin-4-yl)urea (TPPU) is a soluble epoxide hydrolase (sEH) inhibitor that has been reported to prevent glial cell reactivation and alleviate cognitive deficits in the brains of AD mice (Ghosh et al., 2020). Rapamycin is a potential small molecule that enhances autophagy in AD and accelerates the clearance of necrotic nerves (Majumder et al., 2011). Lin et al. developed platelet cell membrane liposomes carrying TPPU and rapamycin (TR@CPL) (Lin et al., 2024). Three months after TR@CPL injection into mice through the tail vein, there was less glial infiltration and reduced neuroinflammation. The transcription factor PU.1 is highly expressed in microglia and is positively correlated with the development of AD (Penney et al., 2020). Lowering PU.1 expression is considered an effective way to protect against AD (Pimenova et al., 2021). RNA therapeutics can ameliorate AD-associated transcriptional and translational changes in microglia. To increase siRNA protection and BBB permeability, William et al. utilized lipid nanoparticles to deliver PU.1 siRNAs (Ralvenius et al., 2024). Local intrathecal injection of liposomal nanoparticles enhances mRNA delivery within the mouse brain, achieving up to 92% ± 2% PU.1 transcriptional silencing within 12 h in mice. Thereby reducing the expression of neuroinflammatory factors in mice.

Aβ vaccines reduce the long-term accumulation of Aβ by inducing the production of polyclonal anti-Aβ antibodies (Muhs et al., 2007). Regulatory T cells (Tregs) can attenuate neuroinflammation in AD by secreting anti-inflammatory cytokines such as transforming growth factor-β (TGF-β) and interleukin (IL)-10 (Kang et al., 2019). Zheng et al. developed therapeutic nanovaccine lipid nanoparticles (LNP-R/Aβs) loaded with rapamycin and Aβ (Jung et al., 2023). The nanovaccine efficiently delivers rapamycin and Aβ peptides to dendritic cells, produces anti-Aβ antibodies and Aβ-specific Treg cells, alleviates neuroinflammation, and inhibits cognitive impairment in mice. The size of LNP-R/Aβ is 232.7 ± 84.0 nm, and the zeta potential is 9.28 ± 4.74 mV. LNP-R/Aβ accumulates in the lymph nodes of mice 1 day after injection. In mice, LNP-R/Aβ treatment was confirmed to induce Aβ-specific Treg cells in AD, as high levels of Treg cell markers (Foxp3) and anti-inflammatory cytokines (TGF-β and IL-10) were detected, significantly reducing the number of Aβ plaques. However, the Aβ vaccine has achieved some efficacy in preclinical studies. However, United States FDA approval is controversial because the therapeutic effects on cognitive ability barely meet clinical endpoints and are limited to specific patient subpopulations. Therefore, Aβ vaccines still need to address a series of issues before they can be applied for clinical treatment.

### 3.3 Antioxidants

Aβ itself can induce oxidative stress, which also leads to increased production of Aβ (Miller et al., 2009). ROS are products of oxidative stress, which can cause mitochondrial dysfunction and induce neuroinflammation to play an important role in the development of AD (Bai et al., 2022). Elevated ROS concentrations promote tau phosphorylation. Tau protein is hyperphosphorylated, affecting mitochondrial function, causing synaptic dysfunction and even neuronal death which leads to decreased synaptic function, leading to apoptosis and subsequent neurodegeneration (Sen et al., 2018; Mashal et al., 2022). Studies have shown that elevated ROS often precedes Aβ deposition and neurofibrillary tangles (Butterfield and Boyd-Kimball, 2018). Lowering ROS is essential for slowing neurodegeneration in the brains of AD patients (Ionescu-Tucker and Cotman, 2021). Resveratrol (3,5,40-trihydroxystilbene, RES) has potential in the treatment of AD because of its anti-inflammatory, oxidative stress and immunomodulatory effects (Moussa et al., 2017). RES is poorly soluble in water and has low bioavailability, so it is difficult for RES to cross the BBB (Zhang et al., 2021). Cerium oxides (CeO2 and Ce2O3) have excellent biocompatibility and have antioxidant capacity (Bao et al., 2018). Yu et al. synthesized manganese-doped CeO2-doped mesoporous hollow nanoparticle (LMC-RES)-supported RES for the removal of ROS in an AD environment (Hu et al., 2023). The particle size of LMC-RES is approximately 120 nm, and the drug loading for RES is 39.8% ± 3.65. LMC-RES had a good sustained-release effect and good biocompatibility, and the drug release rate of LMC-RES at 24 h was 80.9% ± 2.25%. In a rat model of AD, LMC-RES can cross the BBB and be enriched in neurons, inhibiting oxidative stress and reducing ROS production through the Nrf-2/HO-1 signaling pathway.

The ability of Prussian blue (PB) to scavenge ROS is attributed to its affinity for hydroxyl radicals (Zhang et al., 2016). The ability of PB to scavenge ROS effectively has a potential neuroprotective effect on AD (Zhang et al., 2016). Poly(amide amine) (PAMAM) dendritic polymers can enhance the ability of PB to penetrate the BBB. Angiopep-2 (ANG-2), a ligand that targets low-density lipoprotein receptor-associated protein-1 (LPR-1) on the surface of cerebral capillary endothelial cells, can be coupled to functional groups on the surface of PAMAM to enhance the targeting of nanoparticles (Zou et al., 2020). Zhong et al. combined ANG-2-modified PAMAMs with PB nanoparticles to make PPA NPs improve the BBB transport efficiency of PB (Zhong et al., 2022). PPA NPs have a zeta potential of +19 mV and are noncytotoxic in mice. PPA NPs can be deposited in brain tissue via the BBB after 4 h of injection into mice via the tail vein. PPA NPs are able to activate mitophagy to eliminate abnormal mitochondria that produce large amounts of ROS. Breakthroughs have been made in the antioxidant nature of metal nanoparticles and their oxides, such as zinc oxide nanoparticles (El-Sayed et al., 2023). Zinc ions can reduce the incidence of infections as well as the production of inflammatory cytokines (Hussein et al., 2022). El-Sayed et al. reported that zinc oxide nanoparticles have a unique effect on acetylcholinesterase inhibition (El-Sayed et al., 2020). Elmonem et al. reported that zinc oxide nanoparticles ameliorate the neurotoxic effects of AD by decreasing IL-1β, TNF-α, and acetylcholinesterase activity and increasing glutathione production (Abd Elmonem et al., 2023).

Curcumin can activate Akt phosphorylation, which reduces Aβ production and plaque deposition, downregulate the production of AD-related cytokines (Ege, 2021). The poor bioavailability of curcumin limits its clinical application because of the difficulty of passing through the BBB (Xia et al., 2023a). PLGA is a copolymer with excellent biocompatibility and biodegradability (Xia Y. L. et al., 2022). Anila et al. developed curcumin-encapsulated PLGA nanoparticles for the treatment of AD (Mathew et al., 2012). PLGA-curcumin nanoparticles have particle sizes between 150 and 200 nm and zeta potentials in the range of −30 to −20 mV. PLGA-curcumin nanoparticles have nearly 60% free radical scavenging activity. Quercetin (QT) inhibits AchE, which prevents acetylcholine degradation and leads to a decrease in Aβ aggregate production that mitigates AD progression (Khan et al., 2020). QT is poorly bioavailable and often requires large doses to be effective (Ratnam et al., 2006). Sun et al. developed PLGA nanoparticles (PLGA@QT NPs) for QT encapsulation for the treatment of AD (Sun et al., 2016). PLGA@QT NPs have an average diameter in the range of approximately 100–150 nm and a QT loading rate of 35%. *In vitro*, the NPs were shown to release 100% of the QT within 2 h of PLGA@QT NP treatment. In mice, PLGA@QT NPs were shown to delay the progression of AD.

### 3.4 Induction of nerve repair

Nerve growth factor (NGF) promotes cranial nerve repair, prevents nerve damage and restores nerve cell function (Xia et al., 2023b). NGF is widely used in neuroregenerative diseases (Wang et al., 2022). However, NGF alone is readily degraded by biological enzymes during delivery and has difficulty crossing the blood‒brain barrier (BBB) to reach the lesion to function (Kuo et al., 2017). The brain-targeted peptide RVG has been widely used to improve the BBB penetration efficiency of drugs. Yuan et al. utilized a diselenide-rich ROS-responsive nanoplatform (R@NGF-Se-Se-Ru) drug delivery system to deliver NGF (Yuan et al., 2021). Under near-infrared (NIR) irradiation, R@NGF-Se-Se-Ru has good photothermal properties and decomposes nanoclusters into small nanoparticles. Due to the targeting ability of RVG, these nanoparticles can penetrate the BBB, which can effectively inhibit the aggregation of Aβ and promote neurite outgrowth, thereby delaying the progression of AD. R@NGF-Se-Se-Ru has an average particle size of 80 nm and loads of 22.2 and 13 mmol/mg for RVG and NGF, respectively. Some degree of neuronal recovery was detected after the R@NGF-Se-Se-Ru tail vein was injected into the mice, which were then subjected to NIR irradiation for 4 days. Metal‒organic frameworks (MOFs) have emerged as drug carriers for the delivery of organic molecules, biomacromolecules, and nanoparticles (Chen et al., 2018). Yu et al. developed nanoparticles using the antioxidant cerium for codelivery of siSOX9 and retinoic acid (RA) into NSCs for nerve repair in AD (Yu et al., 2020). The SOX9 protein is an essential transcription factor that benefits gliogenesis, and siSOX9 can downregulate the expression of the SOX9 protein (Doerks et al., 2002). RA has the ability to upregulate neuronal gene expression. The diameter of the nanocomposite is approximately 100–200 nm, and the loading efficiency of siSOX9 is as high as 90.1% (Doerks et al., 2002). *In vitro* use confirmed that 40% of the RA was released from the nanoparticles within 5 h. Cerium dioxide nanoparticles can act as ROS scavengers and can significantly reduce the intracellular levels of ROS. At week 5 in the annotated mice, the nanocomplexes effectively promoted the differentiation of NSCs into neurons and led to alleviation of oxidative stress, decreased neuronal apoptosis, and increased neurite length (Doerks et al., 2002).

Huang et al. genetically engineered neural stem cells (NSCs) to stably express the Aβ-degrading protease NEP, which is expressed on the cell membrane of NSCs and released into exosomes (Huang et al., 2021). To increase muscle delivery efficiency, Huang et al. designed a highly efficient gene and drug delivery nanopreparation (PPAR-siSOX9) using PBAE-PLGA to improve the neuronal differentiation efficiency of NEP-NSCs in the pathological AD microenvironment. Decreased SOX9 expression inhibits the degradation of β-catenin in cells, which further activates the Wnt/β-catenin pathway and promotes the differentiation of NSCs into neurons (Iwata et al., 2000). The nanoformulation has a spherical morphology with a diameter of ≈150 nm and a zeta potential of +23.6 mV (Iwata et al., 2000). Compared with the blank control group, the nanopreparative group differentiated and produced more neurons (69.4%) after 10 days *in vitro*. In one-month-old mice, nanopreparation significantly improved cognitive function to a greater degree (Iwata et al., 2000).

Naringenin is a ketone widely found in fruits, which has antioxidant, free radical scavenging, anti-cancer, and anti-inflammatory effects (Motallebi et al., 2022). Fakhrul et al. demonstrated that Naringenin has a neuroprotective effect and can treat cognitive impairment in AD (Khan et al., 2012). Shadab et al. developed the naringenin nanoemulsion with Naringenin (Md et al., 2018). The study showed that the nanoemulsion formulation loaded with naringenin had a droplet size of 113.83 nm. In mice, it was confirmed that naringenin nanoemulsion loaded with Naringenin significantly reduced Aβ-induced neurotoxicity and effectively induced nerve repair. Li et al. designed a composite nanomaterial of Prussian blue nanoparticles (PB NPs/MM) encapsulated in macrophage membranes (Li et al., 2025). Macrophage membranes have stronger targeting capabilities and more efficient immune evasion than nanoliposomes (Xi et al., 2022). The excellent photothermal capability of PB NPs can briefly open the BBB under near-infrared laser irradiation, thereby enhancing the transmission efficiency of PB NPs/MM in the BBB and ablating Aβ deposits (Liu et al., 2021). Under the condition of photothermal treatment, PB NPs/MM not only cleared the surrounding inflammation by the enrichment of the lesion site, but also promoted the nerve repair function of the mouse model of advanced AD.

## 4 Facing challenges

Neurological symptoms due to AD continue to be a challenge for clinicians. Drug therapy has controlled the progression of AD to some extent (Pena et al., 2020). Drug delivery to the brain remains a challenge because of the presence of the BBB, lipophilicity, high molecular weight of the drug, and other factors. Therefore, nanoparticle-mediated delivery of targeted drugs to the brain to delay AD progression has been explored in recent years. As a promising strategy, nanoparticles can reduce enzymatic degradation, endothelial cell clearance, and peripheral side effects while improving targeting and bioavailability and helping to overcome BBB barriers (Liu et al., 2023). Despite these potential advantages, there are still some challenging aspects of nanocarrier-mediated drug delivery, such as the toxicity of nanoparticles (Lamptey et al., 2022). The toxicity of nanoparticles depends mainly on size, surface charge, ion dissolution, and shape (Zielińska et al., 2020). First, choosing the optimal NP size for AD treatment is difficult. Studies have shown that nanoparticles with a particle size between 50 and 100 nm are more suitable for the treatment of AD, and the larger the size is, the lower the drug loading and BBB permeability. The smaller the size is, the greater the accumulation and toxicity (Liu and Shen, 2023; Sonavane et al., 2008). Rafael et al. demonstrated in the study of nanoparticle-targeted AD that smaller and negatively charged NPs can effectively cross the BBB and exhibit more neurosuppressive effects (Martín-Rapun et al., 2017). Jasjeet et al., in a review of nanoparticles on AD, concluded that the toxicity of naturally loaded nanoparticles is greatly reduced, this has only been reported in preclinical studies, and further verification of their toxicity *in vivo* is still needed (Sahni et al., 2011). In addition, the surface charge, ion dissolution, and shape of nanoparticles also affect their targeting and drug-loaded pharmacokinetics (Iqbal et al., 2024). Although improved nanoparticles can remedy best problems, their safety *in vivo* has not been clearly verified (Bi et al., 2024). Although nanoparticles have achieved good results in preclinical studies, there are some challenges to their clinical translation. There are still no clinical trials of nanoparticles for the treatment of AD.

## 5 Conclusion

AD is one of the most dangerous diseases that threatens the physical and mental health of older adults. As the world’s population ages, the number of older people is increasing. However, existing treatment strategies are far from satisfying the need for treatment by merely improving symptoms. The presence of the BBB prevents most drugs from penetrating the brain to exert their effects. The most important strategy for AD treatment is to develop effective drugs that can work in the brain through the BBB. Over the past few decades, therapeutic nanomedicine has also begun to gain momentum in the field of neuroscience. Owing to their natural advantages, nanoparticles have the potential to achieve targeted intracerebral drug delivery for the diagnosis, prevention, and treatment of neurological diseases. Nanomaterials can be improved in a controlled manner through internal and surface chemical modifications, with increased drug loading efficiency and targeted function, which can safely and effectively deliver loaded drugs to the lesion site. In addition, nanomaterials can provide new ideas for the treatment of AD through anti-aging, anti-neuroinflammatory, antioxidative, and nerve repair effects. However, the use of nanoparticles for clinical applications in the treatment of AD has rarely been reported. Medical protocols for the approval and validation of brain-targeted nanomedicines are still in their infancy. The clinical translation of nanoparticles faces some challenges, such as nanotoxicity, safe production and other factors. Although most nanoreports exist only in preclinical studies, we believe that more nanoparticle-based AD therapies are expected to be available in the near future.
